# Effects of Mindfulness-Based Stress Reduction Combined With Perioperative Refined Nursing on Postoperative Sleep Disturbances, Anxiety, and Depression in Patients Undergoing Coronary Artery Bypass Grafting

**DOI:** 10.62641/aep.2291

**Published:** 2026-10-15

**Authors:** Huajun Yao, Caihong Peng, Min Wu

**Affiliations:** ^1^Department of Nursing, Ruijin Hospital, Shanghai Jiao Tong University School of Medicine, 200025 Shanghai, China; ^2^Department of Cardiac Surgery, Ruijin Hospital, Shanghai Jiao Tong University School of Medicine, 200025 Shanghai, China

**Keywords:** mindfulness-based stress reduction, perioperative refined nursing, coronary artery bypass grafting, sleep disturbances, negative emotion

## Abstract

**Background:**

To assess whether integrating mindfulness-based stress reduction (MBSR) into refined perioperative nursing is associated with improvements in postoperative sleep disturbances, anxiety, and depression in patients undergoing coronary artery bypass grafting (CABG).

**Methods:**

We retrospectively reviewed 146 CABG patients treated at Ruijin Hospital, Shanghai Jiao Tong University School of Medicine, from July 2023 to August 2025. To reduce selection bias, 1:1 propensity score matching (PSM) was performed based on baseline characteristics, yielding 73 matched pairs for analysis. Based on the nursing protocol documented in the medical records, patients were classified into two groups: a standard care group had received standard refined perioperative nursing, while an MBSR group had received the same care supplemented with MBSR. Outcome measures included Hospital Anxiety and Depression Scale (HADS) scores assessed preoperatively and on postoperative day 7, objective sleep architecture indices obtained via actigraphy on the day of surgery and postoperative day 7, Pittsburgh Sleep Quality Index (PSQI) scores collected within 24 hours after surgery and on postoperative day 7, length of hospital stay, time to first postoperative ambulation, incidence of sleep disturbances on postoperative day 7, and serum levels of interleukin-6 (IL-6), cortisol, orexin, and tumor necrosis factor-α (TNF-α) measured preoperatively and at 24 and 72 hours postoperatively. Statistical analyses included t-tests, repeated measures analysis of variance, and chi‑square tests.

**Results:**

After PSM, 73 patients were included in each group, with all standardized mean differences <0.1. Baseline preoperative HADS scores did not differ between groups (*p* > 0.05). On postoperative day 7, both groups showed reductions in (HADS-Anxiety) HADS-A and HADS-Depression (HADS-D), with the MBSR group exhibiting significantly lower scores on all HADS subscales than the standard care group (*p* < 0.05). PSQI scores on the day of surgery were similar (*p* > 0.05); On postoperative day 7, both groups improved in total PSQI and across its seven domains versus the surgical day. The MBSR group had significantly better scores than the standard care group for all PSQI domains and total score except subjective sleep quality and hypnotic use (*p* < 0.05). Time to first postoperative ambulation was shorter and the rate of sleep disturbance on postoperative day 7 was lower in the MBSR group (*p* < 0.05); mean hospital stay did not differ (*p* > 0.05). Objective sleep parameters on the day of surgery were comparable (*p* > 0.05); on postoperative day 7 both groups showed increased total sleep time and sleep efficiency, with greater gains in the MBSR group (*p* < 0.05). Nighttime awakenings decreased in both groups, with the MBSR group showing a larger reduction (*p* < 0.05). Repeated-measures analysis of variance (ANOVA) revealed significant effects of time, group, and time-by-group interaction for cortisol, orexin, IL-6, and TNF-α (*p* < 0.05). Within-group analyses showed postoperative increases in cortisol, IL-6, and TNF-α at 24 and 72 hours and declines in orexin versus baseline (*p* < 0.05). Between-group comparisons at 24 and 72 hours postoperatively demonstrated lower cortisol, IL-6, and TNF-α and higher orexin in the MBSR group compared with the standard care group (*p* < 0.05), and the temporal patterns differed significantly between groups (*p* < 0.05).

**Conclusions:**

Refined perioperative nursing incorporating MBSR was associated with alleviation of negative emotions in patients undergoing CABG, a lower incidence of sleep disturbances on the 7th postoperative day, and shorter time to first postoperative ambulation.

## Introduction

Coronary artery bypass grafting (CABG) is a crucial revascularization strategy for end-stage coronary artery disease. Patients experience high perioperative stress, and postoperative sleep disruption often persists throughout monitoring, analgesia, and functional recovery. Disrupted sleep architecture can impair tissue repair and immune homeostasis. Furthermore, via activation of the hypothalamic-pituitary-adrenal (HPA) axis and sympathetic nervous system, it can amplify inflammatory responses, which are associated with delirium, myocardial ischemic events, and readmission risk [1]. Concurrently, anxiety, depression and sleep disturbances often exacerbate each other, creating a vicious cycle of psychological stress and physiological dysregulation that is difficult to ameliorate solely with analgesia, sedation, or general health education [2, 3]. Current perioperative nursing practices primarily emphasize procedural standardization and safety checks, often lacking proactive identification and management of psychological stressors, process modulation, and guidance for postoperative sleep. Preoperative visits tend to focus on information delivery, with insufficient structured catastrophic thinking and avoidance behaviors [4]. Mindfulness-based stress reduction (MBSR) has shown efficacy in improving negative emotions and stress responses in populations with coronary artery disease by cultivating non-judgmental awareness and acceptance. However, the traditional MBSR course duration may not align well with the perioperative timeline, and its integration pathways into clinical scenarios require further validation [5]. Based on previous clinical nursing records, this study analyzed the integration of MBSR-related nursing elements into the refined perioperative nursing process and their association with sleep outcomes, negative emotions, and stress-inflammatory indicators, using propensity score matching (PSM) to reduce bias from baseline differences, thereby providing a reference for optimizing perioperative psychological and behavioral nursing models.

## Materials and Methods

### General Information

#### Case Data

A total of 358 patients who underwent CABG at Ruijin Hospital, Shanghai Jiao Tong University School of Medicine between July 2023 and August 2025 were initially screened.

Inclusion criteria: (1) First-time CABG surgery; (2) Age ≥18 and ≤85 years, gender unrestricted; (3) Relatively complete medical records allowing clear distinction between receiving “MBSR-integrated perioperative refined nursing” or “standard perioperative refined nursing”; (4) Preoperative Pittsburgh Sleep Quality Index (PSQI) score ≤7 [6]; (5) Complete clinical and follow-up data available for obtaining required baseline and outcome measures.

Exclusion criteria: (1) Emergency surgery, redo cardiac surgery, or concurrent other major cardiac surgery; (2) Pre-existing diagnosis of sleep apnea syndrome or other known severe sleep disorders; (3) Long-term regular use of antipsychotics or sedative-hypnotics; (4) History of diagnosed severe psychiatric illness; (5) Postoperative severe complications such as delirium or cerebrovascular accident; (6) Receipt of systematic psychotherapy affecting sleep during the postoperative assessment period.

After applying inclusion and exclusion criteria, 174 were excluded: 36 for non-first CABG, 8 for age <18 or >85 years, 24 for emergency or redo cardiac surgery or concurrent major cardiac procedures, 42 for preoperative PSQI score >7, 16 for incomplete clinical or follow-up data, 14 for pre-existing diagnosed sleep apnea syndrome or severe sleep disorders, 18 for long-term regular use of antipsychotics or sedative-hypnotics, 6 for history of diagnosed severe psychiatric illness, and 10 for severe postoperative complications such as delirium or cerebrovascular accident. Thus, 184 eligible patients remained.

Based on the nursing protocol recorded in the medical records, 102 had received standard refined perioperative nursing (standard care group) and 82 had received the same nursing plus MBSR (MBSR group). To control confounding factors, 1:1 PSM using nearest-neighbor matching with a caliper width of 0.2 standard deviations of the logit of the propensity score was performed. Matching variables included age, gender, body mass index (BMI), New York Heart Association functional classification (NYHA) class [7], surgical approach, cardiovascular risk factors (hypertension, hyperlipidemia, diabetes, smoking history), left ventricular ejection fraction (LVEF), operative time, and education level. After matching, 73 patients were successfully paired in each group, with all standardized mean differences (SMD) <0.1, resulting in a final analytical sample of 146 patients. The study was performed in accordance with the Declaration of Helsinki and was approved by the Ethics Committee of Ruijin Hospital, Shanghai Jiao Tong University School of Medicine (Approval No.: (2025) Clinical Research Ethics Review No. (263)). Informed consent was obtained from all participants involved in the study.

### Methods

The nursing protocols were retrospectively extracted from electronic medical records, operating room nursing records, preoperative visit records, handover records, and ward nursing notes; the researchers did not reassign or implement nursing interventions during the study period. All descriptions below represent the care activities as documented in these records.

Standard care group: (1) Preoperative Visit and Assessment: According to the records, the circulating nurse visited the ward on the afternoon before surgery with the surgical notification, reviewed the electronic medical record to verify patient identity, surgical site, anesthesia type, and preoperative order compliance. Using a standardized preoperative checklist, vital signs, skin integrity, venous access, allergies, and past medical history were documented as having been assessed. Key checks included confirming appropriate discontinuation of anticoagulants per anesthesia requirements and availability of all preoperative reports. The nurse documented that the operating room layout, admission process, anesthesia induction, and postoperative awakening were explained to the patient and family, emphasizing NPO times (8h for solids, 2h for clear liquids) and the purpose of potential intraoperative lines. The visit was recorded as lasting 15-20 minutes, after which the form was archived. (2) Intraoperative Standardized Positioning: After anesthesia induction, the circulating nurse and surgeon confirmed the surgical position. Gel head rings, positioning pads, and restraints were used. Pressure injury prevention measures included silicone dressings on bony prominences and avoiding defibrillation areas for electrodes. Skin color and temperature at pressure points were documented as checked every 30 minutes, adjusting limbs to maintain functional alignment. Non-operative areas were covered with warming blankets to maintain core temperature ≥36°C. The scrub nurse scrubbed in 30 minutes early. Together with the circulating nurse, they performed an initial count of instruments, sponges, and needles using a two-person read-aloud method documented on the count sheet. During surgery, sterile fields were kept dry, soaked drapes were replaced promptly, and instrument transfer followed no-touch technique guidelines. (3) Postoperative Transfer and Handover: Per the records, after extubation, transfer proceeded only when the anesthesiologist assessed a Steward Recovery Score [8]≥4. The circulating nurse connected portable monitoring (ECG, BP, SpO_2_), secured chest tubes, urinary catheter, and IV lines, ensuring drainage bags were below the insertion site. During transfer, the patient’s head was turned to the side to prevent aspiration. Upon intensive care unit (ICU) arrival, an Situation-Background-Assessment-Recommendation (SBAR) [9] handover was conducted, communicating intraoperative details, fluid and blood administration, special medications, skin condition, and tubes. Both nurses signed the handover form after confirmation. Post-return, nursing documentation was completed, ensuring consistency with anesthesia and surgical records. The overall nursing implementation had encompassed the operating room phase (from the afternoon before surgery to completion of post-operative transfer) and the postoperative continuation phase (routine care by the receiving unit according to handover notes up to postoperative day 7).

MBSR group: (1) Mindfulness Introduction and Preoperative Psychological Preparation: Documentation indicated that, on the preoperative afternoon, a senior operating room (OR) nurse trained in MBSR conducted a 25–30 minute one-on-one session in a quiet ward area. It began with 3 minutes of breath awareness, focusing on nasal airflow sensation and abdominal movement. Using the “thoughts are just thoughts” technique from Mindfulness-Based Cognitive Therapy, the nurse was recorded as having helped patients identify automatic negative thoughts about surgery, reframing “surgery is dangerous” to “I notice I’m having a worried thought about surgery.” A pictorial OR environment handbook was provided, encouraging curious observation rather than judgmental reactions. The session concluded by teaching a simplified body scan exercise for practice at bedtime. (2) Intraoperative Mindful Environment and Sensory Management: According to the records, mindfulness began in the preoperative holding area. The nurse provided noise-canceling headphones playing clinically validated mindfulness audio with nature sounds and gentle guidance, encouraging awareness of body-contact, ambient temperature, and breath rhythm. Pre-induction, using the 5-4-3-2-1 grounding technique (naming 5 seen, 4 heard, 3 touched, 2 smelled, 1 tasted item), the nurse guided the patient to reduce anxiety. Low lighting was maintained, unnecessary noise minimized, and silent slide tables used for instrument passing. Before critical steps (e.g., sternotomy, cardiopulmonary bypass), a gentle touch on the forearm served as a non-verbal cue to help anchor attention, coordinating with anesthesia for sedation depth adjustment. (3) Early Postoperative Mindfulness Practice and Sleep Guidance: Upon meeting transfer criteria, the OR nurse was documented as having provided brief mindfulness guidance before handover. Assisting the patient into a semi-Fowler’s position, they guided three rounds of 4-7-8 breathing (inhale 4s, hold 7s, exhale 8s) to alleviate emergence agitation. On postoperative day 1 morning, the ward nurse was recorded as having guided a 10-minute seated meditation, focusing on primary awareness of pain sensations, distinguishing them from secondary emotional reactions. Postoperative sleep hygiene points (e.g., night-time noise/light reduction, use of eye masks/earplugs, minimizing non-essential disruptions) were documented in the handover form as communicated to the receiving unit. (4) Mindful Pain Management and Early Mobilization: Key points for mindful pain coping and early activity were recorded in a written handover addendum for the receiving unit. Strategies involved breath awareness and sensation labeling during pain assessment and initial mobilization. Specifically, the ward nurse was documented as having guided the patient three times daily (8:00, 14:00, 20:00) in labeling pain sensations (e.g., “sharp” “pressure” “throbbing”) rather than judging them as “awful.” For early mobilization, prior to the first attempt, a 5-minute intention-setting exercise was recorded as having been conducted (e.g., “I am preparing to sit up, noticing how my body parts coordinate”). During initial sitting at bedside, the nurse synchronized movement with exhalation (move on exhale, pause on inhale), maintaining SpO_2_ ≥95%. Discomfort during activity was rated (0–10 scale); if >6, activity paused, and 10 minutes of seated meditation followed. (5) Family Synchronized Mindfulness Education and Support: According to the records, on the day before surgery, primary caregivers attended a 30-minute small-group session (≤5 families). They were coached to respond to patient discomfort with statements like “I’m here with you, I notice you’re uncomfortable” after taking three deep breaths, rather than immediately offering solutions. A simple supportive touch technique (placing a hand on the patient’s hand with synchronized breathing) was taught. Communication tips encouraged supportive, present-focused language and positive observation (e.g., “Your breathing seems calmer today”) from family members. A QR code for family mindfulness practice audio was provided to help caregivers maintain their own emotional equilibrium. The overall nursing implementation had encompassed the operating room phase (from the afternoon before surgery to completion of post-operative transfer) and the postoperative continuation phase (routine care by the receiving unit according to handover notes up to postoperative day 7).

### Observation Indicators

#### Baseline Data

Extracted from medical records, anesthesia, and surgical notes within 24h preoperatively. Included age, gender, BMI, NYHA functional class, surgical approach (on-pump/off-pump), cardiovascular risk factors (hypertension, hyperlipidemia, diabetes, smoking history), LVEF, operative time, and education level. Education level was categorized into three mutually exclusive groups, junior high school or below, senior high/vocational school/associate degree and bachelor’s degree or above.

#### Hospital Anxiety and Depression Scale (HADS)

HADS [10]: Extracted from nursing assessment records within 24h preoperatively and between 08:00-12:00 on postoperative day 7. Comprises Anxiety (HADS-A) and Depression (HADS-D) subscales (7 items each, score range 0-21 per subscale). Scores ≤7 indicate no significant anxiety/depression; higher scores indicate greater severity.

#### PSQI

PSQI [11]: Extracted from nursing assessment records within 24 hours after surgery and on postoperative day 7 (08:00–12:00). Includes 19 self-rated and 5 bedpartner-rated items (the latter not scored). The seven scored components comprise subjective sleep quality, sleep latency, sleep duration, habitual sleep efficiency, sleep disturbance, use of sleep medication, and daytime dysfunction; each component is scored from 0 to 3, and the global score ranges from 0 to 21. A global score >7 [6] indicates poor sleep quality, with higher scores indicating worse sleep.

#### Average Hospital Stay, Time to First Postoperative Ambulation, Incidence of Sleep Disorders on Postoperative Day 7

Extracted from medical records for both groups. Sleep disorder diagnosis required meeting Diagnostic and Statistical Manual of Mental Disorders, Fifth Edition (DSM-5) [12] criteria and a PSQI global score >7 [6].

#### Objective Sleep Architecture

Monitored using the ActiGraph wGT3X-BT (ActiGraph, LLC, Pensacola, FL, USA) triaxial accelerometer (30 Hz, ±8g) worn on the non-dominant wrist during two periods: 22:00–06:00 on the night of surgery and 22:00–06:00 on the night corresponding to postoperative day 7 (or the night before discharge if discharged earlier). Data analyzed via ActiLife software (Version 6.13.4, ActiGraph, LLC, Pensacola, FL, USA) using the Cole-Kripke algorithm yielded total sleep time (TST), Sleep Efficiency (SE%), and Wake After Sleep Onset (WASO) times.

#### Laboratory Biomarkers

Peripheral venous blood (5 mL) collected at three time points: preoperative morning (fasting), 24h, and 72h postoperatively. Serum separated by centrifugation (3000 rpm, 10 min, 10 cm radius) and stored at –80°C. Serum levels of cortisol, orexin A, interleukin-6 (IL-6), and tumor necrosis factor-α (TNF-α) were measured using ELISA kits (DRG International (Springfield, NJ, USA) EIA-1887 for cortisol; Cusabio CSB-E08859h (Wuhan, Hubei, China) for orexin A; R&D Systems (Minneapolis, MN, USA) D6050 for IL-6; R&D Systems DTA00D for TNF-α). Absorbance was read at 450 nm using a Thermo Scientific Multiskan FC microplate photometer (Model: Multiskan FC, Cat#: 51119000). Procedures followed manufacturers’ protocols.

### Statistical Analysis

Data analysis used SPSS 26.0 (IBM Corp., Armonk, NY, USA). Normally distributed continuous data (Shapiro-Wilk test) were described as ($\bar{x}$ ± s) deviation. For multi-timepoint comparisons, repeated measures analysis of variance (ANOVA) was applied. Intergroup and intragroup comparisons used independent samples t-test and paired samples t-test, respectively. Non-normally distributed continuous data were described as M (P25, P75), with Mann-Whitney U and Wilcoxon signed-rank tests for intergroup and intragroup comparisons. Categorical data were described as n (%) and compared using chi-square test. Two-sided tests were applied, and *p* < 0.05 was considered statistically significant.

## Results

### Comparison of Baseline Characteristics

Before matching, a significant difference in age was observed between the groups (*p* < 0.05). After matching, no significant differences were found between the standard care group and the MBSR group regarding age, gender, BMI, NYHA class, surgical approach, cardiovascular risk factors, LVEF, operative time, or education level (all *p* > 0.05). See Tables 1 and 2.

**Table 1.** **Comparison of baseline characteristics before matching**.

Baseline characteristic		Standard care group (n = 102)	MBSR group (n = 82)	t/χ^2^	*p*
Gender [n (%)]				3.229	0.072
	Male	68 (66.67)	44 (53.66)		
	Female	34 (33.33)	38 (46.34)		
Age (years)		72.08 ± 5.51	69.95 ± 5.78	2.550	0.012
BMI (kg/m^2^)		24.81 ± 2.63	24.60 ± 2.81	0.522	0.602
NYHA class [n (%)]				2.017	0.365
	Class I	9 (8.82)	9 (10.98)		
	Class II	35 (34.31)	35 (42.68)		
	Class III	58 (56.86)	38 (46.34)		
Surgical approach [n (%)]				0.450	0.502
	Off-pump	61 (59.80)	53 (64.63)		
	On-pump	41 (40.20)	29 (35.37)		
Cardiovascular risk factors [n (%)]					
	Hypertension	80 (78.43)	55 (67.07)	3.001	0.083
	Hyperlipidemia	42 (41.18)	33 (40.24)	0.016	0.898
	Diabetes	40 (39.22)	29 (35.37)	0.288	0.592
	Smoking history	57 (55.88)	43 (52.44)	0.217	0.641
LVEF (%)		52.02 ± 6.89	52.33 ± 7.15	−0.298	0.766
Operative time (min)		254.87 ± 35.42	249.06 ± 38.10	1.069	0.286
Education level [n (%)]				1.065	0.587
	Junior high school or below	27 (26.47)	19 (23.17)		
	Senior high/vocational school/associate degree	42 (41.18)	40 (48.78)		
	Bachelor’s degree or above	33 (32.35)	23 (28.05)		

Note: Data are presented as mean ± standard deviation for continuous variables and as n (%) for categorical variables. Between-group comparisons were performed using independent samples t-test for continuous variables and chi-square (χ^2^) test for categorical variables. BMI, body mass index; NYHA, New York Heart Association functional classification; LVEF, left ventricular ejection fraction; MBSR, mindfulness-based stress reduction.

**Table 2.** **Comparison of baseline characteristics after matching**.

Baseline characteristics		Standard care group (n = 73)	MBSR group (n = 73)	t/χ^2^	*p*
Gender [n (%)]				0.445	0.505
	Male	43 (58.90)	39 (53.42)		
	Female	30 (41.10)	34 (46.58)		
Age (years)		71.14 ± 5.36	70.48 ± 5.89	0.708	0.480
BMI (kg/m^2^)		24.74 ± 2.56	24.69 ± 2.74	0.114	0.909
NYHA class [n (%)]				0.441	0.802
	Class Ⅰ	7 (9.59)	8 (10.96)		
	Class Ⅱ	28 (38.36)	31 (42.47)		
	Class Ⅲ	38 (52.05)	34 (46.58)		
Surgical approach [n (%)]				0.116	0.734
	Off-pump	44 (60.27)	46 (63.01)		
	On-pump	29 (39.73)	27 (36.99)		
Cardiovascular risk factors [n (%)]					
	Hypertension	54 (73.97)	49 (67.12)	0.824	0.364
	Hyperlipidemia	28 (38.36)	30 (41.10)	0.114	0.735
	Diabetes	25 (34.25)	27 (36.98)	0.120	0.730
	Smoking history	36 (49.32)	39 (53.42)	0.247	0.619
LVEF (%)		52.36 ± 6.74	52.19 ± 7.22	0.147	0.883
Operative time (min)		253.23 ± 34.87	249.78 ± 38.44	0.568	0.571
Education level [n (%)]				0.694	0.707
	Junior high or below	19 (26.03)	17 (23.29)		
	Senior high/vocational school/associate degree	31 (42.47)	36 (49.32)		
	Bachelor’s degree or above	23 (31.51)	20 (27.40)		

Note: Data are presented as mean ± standard deviation for continuous variables and as n (%) for categorical variables. Between-group comparisons were performed using independent samples t-test for continuous variables and chi-square (χ^2^) test for categorical variables. BMI, body mass index; NYHA, New York Heart Association functional classification; LVEF, left ventricular ejection fraction; MBSR, mindfulness-based stress reduction.

### Comparison of HADS Scores

Baseline HADS scores did not differ significantly between the two groups (*p* > 0.05). On postoperative day 7, both groups showed reductions in HADS-Anxiety (HADS-A) and HADS-Depression (HADS-D) scores relative to baseline, and the MBSR group had significantly lower scores on each HADS subscale compared with the standard care group (*p* < 0.05). See Table 3.

**Table 3.** **Comparison of HADS scores between groups ($\bar{x}$ ± s, points)**.

Group	n	HADS-A		HADS-D	
		Preoperative	Postoperative day 7	Preoperative	Postoperative day 7
Standard care group	73	10.41 ± 1.41	9.16 ± 1.20^†^	9.45 ± 1.33	8.10 ± 0.96^†^
MBSR group	73	10.36 ± 1.56	8.05 ± 0.94^†^	9.51 ± 1.26	7.74 ± 0.74^†^
t		0.203	6.222	–0.280	2.538
*p*		0.839	<0.001***	0.780	0.012*

Note: Data are presented as mean ± standard deviation ($\bar{x}$ ± s). HADS, Hospital Anxiety and Depression Scale; HADS-A, Anxiety subscale; HADS-D, Depression subscale. *p* values are for between-group comparisons using independent samples t-test. ^†^*p* < 0.05 compared with preoperative values (within-group comparison). *p < 0.05, **p < 0.01, ***p < 0.001 compared with the standard care group at the same time point. MBSR, mindfulness-based stress reduction.

### Comparison of PSQI Scores

PSQI global scores within 24 hours after surgery were comparable between groups (*p* > 0.05). On postoperative day 7, both groups showed significant reductions in global PSQI score and scores across all seven components compared to scores obtained within 24 hours after surgery. The MBSR group had significantly lower scores than the standard care group in all components except sleep quality and use of sleep medication, and also had a significantly lower global PSQI score (*p* < 0.05). See Table 4.

**Table 4.** **Comparison of PSQI scores between the two groups ($\bar{x}$ ± s, points)**.

Dimension	Time	Standard care group (n = 73)	MBSR group (n = 73)	Z/t	*p*
Sleep quality	Within 24 h after surgery	2.0 (1.0, 2.0)	2.0 (1.0, 2.0)	0.129	0.856
	Postoperative day 7	1.0 (1.0, 2.0)^†^	1.0 (1.0, 2.0)^†^	1.229	0.083
Sleep latency	Within 24 h after surgery	2.0 (1.0, 2.0)	2.0 (1.0, 2.0)	–0.362	0.637
	Postoperative day 7	1.0 (1.0, 2.0)^†^	1.0 (1.0, 1.0)^†^	2.699	0.005**
Sleep duration	Within 24 h after surgery	2.0 (1.0, 2.0)	2.0 (1.0, 2.0)	0.331	0.664
	Postoperative day 7	1.0 (1.0, 2.0)^†^	1.0 (1.0, 1.0)^†^	3.218	0.001**
Habitual sleep efficiency	Within 24 h after surgery	2.0 (1.0, 2.0)	2.0 (1.0, 2.0)	–0.072	0.923
	Postoperative day 7	1.0 (1.0, 1.0)^†^	1.0 (1.0, 1.0)^†^	3.853	0.001**
Sleep disturbance	Within 24 h after surgery	2.0 (1.0, 2.0)	2.0 (1.0, 2.0)	–0.037	0.958
	Postoperative day 7	1.0 (1.0, 1.0)^†^	1.0 (1.0, 1.0)^†^	2.112	0.018*
Use of sleep medication	Within 24 h after surgery	1.0 (1.0, 1.0)	1.0 (1.0, 1.0)	–0.057	0.940
	Postoperative day 7	1.0 (1.0, 1.0)^†^	0.0 (0.0, 1.0)^†^	1.426	0.062
Daytime dysfunction	Within 24 h after surgery	1.0 (1.0, 2.0)	1.0 (1.0, 1.0)	0.010	0.987
	Postoperative day 7	1.0 (1.0, 2.0)^†^	1.0 (1.0, 1.0)^†^	2.403	0.009**
PSQI total score	Within 24 h after surgery	10.44 ± 1.56	10.42 ± 1.15	0.088	0.930
	Postoperative day 7	7.93 ± 1.20^†^	6.36 ± 1.20^†^	7.904	<0.001***

Note: PSQI, Pittsburgh Sleep Quality Index; MBSR, mindfulness-based stress reduction. higher scores indicate poorer sleep quality. Total PSQI score data are presented as mean ± standard deviation ($\bar{x}$ ± s) and were compared between groups using independent samples t-test. Individual component scores are presented as median (P25, P75) and were compared between groups using the Mann-Whitney U test. ^†^*p* < 0.05 compared with the within-24-h postoperative assessment within the same group; *p < 0.05, **p < 0.01, ***p < 0.001 compared with the Standard Care group at the same time point.

### Comparison of Average Hospital Stay, Time to First Ambulation, and Incidence of Sleep Disorders on Postoperative Day 7

The MBSR group had a significantly shorter time to first postoperative ambulation and a significantly lower incidence of sleep disorders on postoperative day 7 compared to the standard care group (*p* < 0.05). The average hospital stay did not differ significantly between groups (*p* > 0.05). See Table 5.

**Table 5.** **Comparison of average hospital stay, time of first postoperative ambulation, and incidence of sleep disorders within 7 days after surgery between the two groups**.

Group	n	Average hospital stay (d)	Time to first postoperative	Incidence of sleep disorders on
			ambulation (h)	postoperative day 7 [n (%)]
Standard care group	73	10.24 ± 2.03	28.85 ± 4.74	50 (68.49)
MBSR group	73	9.66 ± 1.89	26.94 ± 4.22	13 (17.81)
t/χ^2^		1.787	2.571	39.509
*p*		0.076	0.011*	<0.001***

Note: MBSR, mindfulness-based stress reduction. Data are presented as mean ± standard deviation for continuous variables and as n (%) for categorical variables. Between-group comparisons were performed using independent samples t-test for continuous variables (average hospital stay and time to first postoperative ambulation) and chi-square (χ^2^) test for categorical variables (incidence of sleep disorders). d, days; h, hours. Sleep disorders were defined according to DSM-5 criteria combined with a PSQI global score >7 [6]. *p < 0.05, ***p < 0.001 compared with the Standard Care group.

### Comparison of Objective Sleep Parameters

Objective sleep parameters on the night of surgery did not differ between groups (*p* > 0.05). On postoperative day 7, both groups showed significant increases in TST and SE%, and a significant decrease in WASO frequency compared to postoperative night values. The MBSR group exhibited significantly higher TST and SE%, and significantly lower WASO frequency than the standard care group on postoperative day 7 (*p* < 0.05). See Table 6.

**Table 6.** **Comparison of two groups of objective sleep structure indicators ($\bar{x}$ ± s)**.

Group	n	Total sleep time (h)		Sleep efficiency (%)		Wake after sleep onset (times)	
		Postoperative night	Postoperative day 7	Postoperative night	Postoperative day 7	Postoperative night	Postoperative day 7
Standard care group	73	6.15 ± 0.63	6.59 ± 0.74^†^	82.52 ± 8.14	85.11 ± 7.26^†^	6.10 ± 0.76	4.89 ± 0.91^†^
MBSR group	73	6.21 ± 0.74	7.17 ± 0.77^†^	82.36 ± 9.02	87.96 ± 7.41^†^	6.04 ± 0.81	2.97 ± 0.63^†^
t		–0.527	–4.640	0.113	–2.347	0.462	14.822
*p*		0.599	<0.001***	0.911	0.020*	0.645	<0.001***

Note: Data are presented as mean ± standard deviation ($\bar{x}$ ± s). Between-group comparisons were performed using independent samples t-test. Objective sleep parameters were monitored using the ActiGraph wGT3X-BT triaxial accelerometer and analyzed via ActiLife software using the Cole-Kripke algorithm. ^†^*p* < 0.05 compared with the Postoperative night (within-group comparison); *p < 0.05, ***p < 0.001 compared with the Standard Care group at the same time point. MBSR, mindfulness-based stress reduction.

### Comparison of Cortisol, Orexin, IL-6, and TNF-α Levels

Repeated-measures ANOVA indicated significant effects of time and group, and a significant time-by-group interaction for cortisol, orexin, IL-6, and TNF-α (all *p* < 0.05). Within each group, cortisol, IL-6, and TNF-α were elevated at 24 h and 72 h after surgery compared with preoperative values, while orexin concentrations were reduced at those postoperative time points (*p* < 0.05).Between groups, the MBSR group showed significantly lower cortisol, IL-6, and TNF-α and higher orexin at 24 h and 72 h postoperatively than the standard care group (*p* < 0.05). The significant interaction terms reflect differing temporal patterns of these biomarkers between the two groups. See Table 7.

**Table 7.** **Comparison of cortisol, orexin, IL-6 and TNF-α between the two groups ($\bar{x}$ ± s)**.

Indicator	Time	Standard care group (n = 73)	MBSR group (n = 73)	F	*p*
Cortisol (nmol/L)	Preoperative	315.56 ± 42.85	320.04 ± 46.41	F_t⁢i⁢m⁢e_ = 1096.27, F_g⁢r⁢o⁢u⁢p_ = 69.44, F_t⁢i⁢m⁢e×g⁢r⁢o⁢u⁢p_ = 57.47	*p *_t⁢i⁢m⁢e_ < 0.001, *p *_g⁢r⁢o⁢u⁢p_ < 0.001, *p *_t⁢i⁢m⁢e×g⁢r⁢o⁢u⁢p_ < 0.001
	Postoperative 24 h	674.66 ± 96.63*	547.87 ± 85.23*^#^		
	Postoperative 72 h	486.96 ± 69.85*	396.63 ± 67.41*^#^		
Orexin (pg/mL)	Preoperative	285.63 ± 35.74	281.66 ± 36.97	F_t⁢i⁢m⁢e_ = 193.91, F_g⁢r⁢o⁢u⁢p_ = 31.93, F_t⁢i⁢m⁢e×g⁢r⁢o⁢u⁢p_ = 25.31	*p *_t⁢i⁢m⁢e_ < 0.001, *p *_g⁢r⁢o⁢u⁢p_ < 0.001, *p *_t⁢i⁢m⁢e×g⁢r⁢o⁢u⁢p_ < 0.001
	Postoperative 24 h	194.41 ± 32.03*	225.54 ± 36.02*^#^		
	Postoperative 72 h	236.56 ± 30.12*	259.47 ± 34.44*^#^		
IL-6 (pg/mL)	Preoperative	9.15 ± 1.96	9.06 ± 2.02	F_t⁢i⁢m⁢e_ = 128.45, F_g⁢r⁢o⁢u⁢p_ = 5.67, F_t⁢i⁢m⁢e×g⁢r⁢o⁢u⁢p_ = 4.31	*p *_t⁢i⁢m⁢e_ < 0.001, *p *_g⁢r⁢o⁢u⁢p_ = 0.019, *p *_t⁢i⁢m⁢e×g⁢r⁢o⁢u⁢p_ = 0.014
	Postoperative 24 h	124.23 ± 25.89*	97.63 ± 24.11*^#^		
	Postoperative 72 h	56.63 ± 14.85*	44.63 ± 12.07*^#^		
TNF-α (pg/mL)	Preoperative	14.96 ± 2.74	15.02 ± 2.55	F_t⁢i⁢m⁢e_ = 1449.01, F_g⁢r⁢o⁢u⁢p_ = 115.95, F_t⁢i⁢m⁢e×g⁢r⁢o⁢u⁢p_ = 78.20	*p *_t⁢i⁢m⁢e_ < 0.001, *p *_g⁢r⁢o⁢u⁢p_ < 0.001, *p *_t⁢i⁢m⁢e×g⁢r⁢o⁢u⁢p_ < 0.001
	Postoperative 24 h	57.85 ± 8.96*	46.77 ± 9.05*^#^		
	Postoperative 72 h	32.12 ± 6.74*	24.55 ± 4.86*^#^		

Note: MBSR, mindfulness-based stress reduction. Data are presented as mean ± standard deviation ($\bar{x}$ ± s). Statistical analysis was performed using repeated measures ANOVA, with F_t⁢i⁢m⁢e_ representing the main effect of time, F_g⁢r⁢o⁢u⁢p_ representing the main effect of group, and F_t⁢i⁢m⁢e×g⁢r⁢o⁢u⁢p_ representing the time-by-group interaction effect. IL-6, interleukin-6; TNF-α, tumor necrosis factor-α. *p < 0.05 compared with Preoperative values (within-group comparison); ^#^*p* < 0.05 compared with the Standard Care group (between-group comparison at the same time point).

## Discussion

As a major means of myocardial revascularization, CABG induces significant physiological and psychological stress responses due to its invasiveness and perioperative demands. High rates of anxiety and depression in the early postoperative period can severely impede cardiac rehabilitation. Previous studies, such as Wei *et al*. [13], Setiawati *et al*. [14] demonstrated that mindfulness meditation effectively reduced anxiety scores and enhanced self-efficacy in CABG patients. A recent meta-analysis further confirmed that mindfulness-based interventions significantly improve anxiety, depression, and stress in patients with coronary artery disease [15]. The present findings align with these reports, showing that patients documented as having received MBSR-integrated perioperative nursing had lower HADS-A and HADS-D scores compared to the standard care group on postoperative day 7. The core mechanism of MBSR involves using “non-judgmental awareness” to interrupt the ruminative cycle of negative thoughts [16]. CABG patients often experience catastrophic thinking regarding surgical outcomes and postoperative pain, leading to sustained sympathetic overactivation [17]. The preoperative mindfulness component documented in this study’s nursing records helped patients recognize “automatic negative thoughts.” The intraoperative use of the 5-4-3-2-1 grounding technique anchored attention to present-moment sensory experience, facilitating cognitive restructuring and preventing the escalation of fear. Additionally, the mindful audio input during surgery may have modulated cortical excitability and reduced amygdala sensitivity to threat stimuli, thereby mitigating emotional stress responses at a neurobiological level [18].

Postoperative sleep disturbance is a common complication following cardiac surgery, influenced by incisional pain, ICU noise, and inflammatory responses, leading to generally poor sleep quality. Safavi *et al*. [19] confirmed that mindfulness training optimizes PSQI scores. Our results showed that while both groups’ PSQI scores improved compared with the within 24 h postoperative assessment, the MBSR group’s scores were significantly better than the standard care group except in sleep quality and sleep medication components on postoperative day 7. This indicates that MBSR-integrated care does not completely reverse postoperative sleep decline but effectively mitigates its severity. The mechanism may involve reduced sleep effort through practices like body scan and breath awareness. Patients under routine care often develop secondary anxiety from striving to sleep, which increases cortical arousal. In contrast, the MBSR group patients were taught to accept present-moment bodily sensations including pain or discomfort, reducing cognitive resistance to the sleep environment. Furthermore, integrating mindfulness principles into OR environment management and documenting postoperative sleep hygiene tips (e.g., noise/light reduction) in the handover form to alert the receiving unit created a synergistic effect between internal psychological steadiness and external stimulus reduction, thereby preserving relatively better sleep quality amidst perioperative stress [20].

Early ambulation is crucial for preventing pulmonary complications and deep vein thrombosis post-CABG, but pain and fear-avoidance behaviors often limit patient willingness. This study found that the MBSR group ambulated earlier and had a lower incidence of sleep disorders by day 7. However, the average hospital stay did not differ significantly, possibly due to fixed discharge criteria within clinical pathways. The earlier ambulation in the MBSR group can be attributed to mindful pain management, which reframes pain from an emotional “suffering” to neutral sensory signals, potentially increasing pain tolerance. The intention-setting and breath-synchronized guidance before activity may have enhanced patients’ self-efficacy and confidence in body control, enabling them to overcome fear and participate actively in early rehabilitation. This positive behavioral pattern likely contributed to circadian rhythm re-establishment, thereby reducing sleep disorder risk [21, 22].

Regarding sleep architecture, studies like Gherardi-Donato *et al*. [22] found that 8-week mindfulness training effectively regulated chronic stress biomarkers and improved mental health indicators, while Spruill *et al*. [23] noted adherence to mindfulness practice correlated with improved psychological outcomes post-MI. Our actigraphy data revealed that on postoperative day 7, both groups had increased TST and SE, with the MBSR group outperforming the standard care group. WASO frequency decreased in both groups, again more so in the MBSR group. The physiological mechanism may involve inhibition of hyperarousal states. Post-CABG patients are often hypervigilant, with minor stimuli triggering micro-arousals and sleep fragmentation. Mindfulness training cultivates focused attention and an accepting attitude, potentially helping patients return to sleep more quickly after nocturnal awakenings instead of engaging in anxiety-driven rumination about insomnia. This psychological regulation may reduce the frequency and duration of awakenings, preserving more consolidated sleep architecture conducive to neural repair and metabolic balance during deep sleep [24]. It should be noted that actigraphy, while advantageous for continuous, non-invasive monitoring in the ward, relies on limb movement to infer sleep status. In postoperative CABG patients, pain, positional restrictions, monitoring devices, and sedative/analgesic medications may affect movement, potentially leading to overestimation of sleep during quiet wakefulness. Therefore, the TST, SE, and awakenings reported here serve as objective references for sleep-activity rhythm but cannot fully replace polysomnography in evaluating sleep stages and architecture. Future studies incorporating PSG are warranted to verify these findings.

Surgical trauma activates the HPA axis and sympathetic nervous system, leading to cortisol and catecholamine release. This, via glucocorticoid receptor signaling, can activate NF-κB, driving a cascade of inflammatory cytokines like IL-6 and TNF-α [25, 26, 27]. Our results showed that at 24h and 72h postoperatively, the MBSR group had lower levels of cortisol, IL-6, and TNF-α, and higher levels of orexin compared to the standard care group. This suggests mindfulness modulates homeostasis through unified neuro-endocrine pathways. The core mechanism may involve enhanced prefrontal cortical regulation over the amygdala, reducing the brain’s sensitivity to threat and thus buffering excessive HPA axis and sympathetic activation at the source [28]. This central regulation has a downstream effect: Reduced stress hormone secretion directly attenuates peripheral inflammatory amplification. Maintaining orexin levels may help counteract postoperative oversedation and inflammatory fatigue, supporting the stabilization of sleep-wake cycles [29].

This study has several limitations. It is a single-center retrospective observational study; case selection, nursing protocol allocation, and data completeness may introduce selection bias. Although PSM was used to balance some baseline characteristics, residual confounding from unmeasured factors such as pain intensity, analgesic/sedative use, nighttime nursing frequency, ward noise, family support, nurse experience, and patient adherence to mindfulness practice cannot be excluded. Additionally, while the standard care group received refined perioperative nursing, there may have been differences in contact time, emotional support intensity, and interaction frequency compared to the MBSR group, potentially inducing a Hawthorne effect from the amount of nursing attention itself, which could influence emotional and sleep outcomes. The use of actigraphy has inherent limitations compared to polysomnography, as detailed above. Moreover, although improvements in sleep, emotions, and some stress-inflammatory markers were observed in the MBSR group, the study design limits causal inference; the interpretations involving HPA axis, inflammation, and neurocognitive pathways are largely based on previous literature and the trends observed herein. Multicenter prospective randomized controlled trials incorporating polysomnography, neurofunctional assessments, and long-term follow-up are needed for further validation.

## Conclusions

In this retrospective analysis of patients undergoing CABG, the integration of MBSR into a refined perioperative nursing protocol was associated with significant alleviation of postoperative anxiety and depression, a lower incidence of sleep disturbances on the seventh postoperative day, and a shorter time to first ambulation. These clinical improvements were paralleled by favorable objective changes in sleep architecture and reductions in stress-inflammatory biomarkers. The findings underscore the potential value of embedding structured psychological support into standard perioperative care pathways to mitigate the neuroendocrine and behavioral consequences of surgical stress.

## Availability of Data and Materials

The data supporting the findings of this study are available from the corresponding author upon reasonable request. These data were extracted from the hospital medical record system.
